# Intimate partner violence perpetration and mental health service use in England: analysis of nationally representative survey data

**DOI:** 10.1192/bjo.2023.51

**Published:** 2023-04-12

**Authors:** Vishal Bhavsar, Sally McManus, Katherine Saunders, Louise M. Howard

**Affiliations:** Section of Women's Mental Health, King's College London, UK; and NIHR Biomedical Research Centre, South London and Maudsley NHS Foundation Trust, London, UK; Violence and Society Centre, City University of London, UK; and National Centre for Social Research, London, UK

**Keywords:** trauma, risk assessment, epidemiology, forensic mental health services, community mental health teams

## Abstract

**Background:**

Intimate partner violence perpetration (IPVP) is associated with psychiatric disorders, but an association with mental health service use has not been fully established and is relevant for policy. Mental health service contact by perpetrators of intimate partner violence presents an opportunity for reducing harmful behaviours.

**Aims:**

To examine the association between IPVP and mental health service use.

**Method:**

Analysis of national probability sample data from the 2014 Adult Psychiatric Morbidity Survey, testing for associations between lifetime IPVP and mental health service use. We assessed the impact of missing data with multiple imputation and examined misreporting using probabilistic bias analysis.

**Results:**

The prevalence of reported lifetime IPVP was similar for men (8.0%) and women (8.6%). Before adjustments, IPVP was associated with mental health service use (odds ratio (OR) for any mental health service use in the past year for men: 2.8 (95% CI: 1.8–4.2), for women: 2.8 (95% CI: 2.1–3.8)). Adjustments for intimate partner violence victimisation and other life adversities had an attenuative influence. Associations remained on restricting comparisons with those without criminal justice involvement (OR for any mental health service use in the past year for men: 2.9 (95% CI: 1.7–4.8), for women: 2.3 (95% CI: 1.7–3.2)).

**Conclusion:**

The strong association of IPVP with mental health service use is partly attributable to the concurrent presence of intimate partner violence victimisation and other life adversities. Efforts to improve the identification and assessment of IPVP in mental health services could benefit population health.

## Intimate partner violence and intimate partner violence perpetration

Intimate partner violence (IPV) is defined by the World Health Organization as behaviour within an intimate relationship which includes physical violence, sexual violence, emotional and/or psychological abuse, and controlling behaviours.^1^ IPV is a persisting public health problem resulting in a large and preventable burden of mortality, morbidity and suffering.^2^ Reducing IPV is a critical policy goal. Criminal justice efforts to respond to IPV perpetration (IPVP) rely on adequate criminal justice identification and/or detection of IPVP.^3^ The UK Home Office estimates that 17% of domestic abuse (which includes IPV) is reported to the police. Alternative or parallel strategies to enhance accurate identification and assessment of IPVP are needed.

There are consistent risk factors for IPVP. IPVP is gendered, with higher prevalence of IPVP among men compared with women^4^ (although not in all studies^5^) and men perpetrating more frequent and severe violence.^6^ IPVP is more common among those with hazardous alcohol and substance use,^7,8^ and among people with other forms of previous offending behaviour (e.g. criminal justice involvement^9^). IPVP is more common in those who are also victims of IPV^10^ and in those experiencing other life adversity (including child maltreatment^11^). IPVP is more frequent among unemployed individuals compared with employed individuals, and among those with unskilled compared with skilled occupations.^12^

## IPVP and mental health services

IPVP is more common among people with psychiatric conditions than the general population.^13,14^ Yu et al^13^ found higher rates of arrest for IPVP among people with psychiatric conditions including personality and affective disorders. This association remained evident after accounting for familial and genetic risk factors through a sibling comparison design. Owing to the association between mental health conditions and IPVP, it has been proposed that health services which provide care for people with mental health conditions (including primary care and secondary mental health services) present opportunities to strengthen identification and assessment of IPVP.^15^ Understanding the distribution of psychiatric morbidity and mental health service use among people reporting IPVP could shape policy by emphasising the role of mental health services as part of a national response to IPVP and guide the development of interventions targeted at specific service settings.

Our aims were to:

- (a) describe associations of lifetime IPVP with use of mental health services in men and women; and
- (b) assess whether these associations are specific to IPVP or a reflection of general violence perpetration.

## Method

### Data collection

The Adult Psychiatric Morbidity Survey (APMS) is a household survey of the general population in England, the last of which was done in 2014.^16^ Briefly, the survey sampled the household residential population of England aged 16 years and above, using a stratified, multistage random sampling design. The sampling frame was based on the national Small User Postcode Address File. The study sample comprised 7546 individuals interviewed at home, representing a response rate of 57%. The initial phase involved computer-assisted personal interviewing, with some sensitive information collected using computer-assisted self-completion interview, in which the participant used the interviewer's laptop.

### Measures

#### Intimate partner violence perpetration

A binary measure of lifetime IPVP was derived based on endorsement of any of the following four items asked in the computer-assisted self-completion interview: have you ever pushed, held or pinned down or slapped a partner or ex-partner; have you ever kicked, bit, hit with a fist or something else, or thrown something at a partner or ex-partner that hurt them; have you ever forced a partner or ex-partner to do something sexual that they didn't want to do; and have you ever frightened a partner or ex-partner by threatening to hurt them or someone close to them?

#### Mental health service use

Participants were asked whether they were currently attending counselling, about any general practitioner (GP) consultation for a physical reason in the past year, any GP consultation for a psychological or emotional problem in the past year, any use of secondary health services (in-patient or out-patient) for a psychological or emotional problem (which we term ‘any use of mental health services’) in the past year and lifetime admission to a psychiatric unit.

#### Sociodemographic characteristics

Age was measured as a continuous variable (in years) during survey data collection. We grouped age into ten-year age bands for descriptive purposes and handled age as a continuous variable in modelling. Educational attainment was grouped into degree-level, teaching/higher national diploma/nursing diploma, A levels, GCSE or equivalent, foreign/other qualifications and no qualifications. Ethnicity was grouped into White British, White Other, Black African or Black Caribbean, Asian or Asian British, and mixed, multiple or other ethnicity. Neighbourhood deprivation was measured according to the English Indices of Deprivation 2010^17^ and grouped into quintiles. Socioeconomic class was measured using the National Statistics Socio-Economic Classification.^18^ To evaluate collinearity between indicators of socioeconomic position (educational attainment, social class and neighbourhood deprivation), we tested correlation among these using Pearson and Spearman correlation indices, setting >0.8 as unacceptable collinearity for modelling.

##### Lifetime IPV victimisation

Lifetime intimate partner violence victimisation was indicated by presence of any of: experience of a partner preventing you from having a fair share of the household money; repeatedly belittling you to the extent that you felt worthless; pushing you, holding you, pinning you down or slapping; sending you more than one unwanted letter, email, text message or card that was either obscene or threatening and which caused you fear, alarm or distress; or kicking you, biting you, hitting you with a fist or something else, or throwing something at you that hurt you.

#### Other life adversities

A scale reflecting the number of other lifetime adversities was created using the List of Threatening Experiences scale,^19^ described as a binary variable cut at the median (three lifetime adverse events) and handled as a continuous variable for modelling. There were lifetime experiences of: serious illness or injury, serious illness or injury to a close relative, serious assault of a close relative, death of an immediate family member, death of a close family friend or other relative, violence at work, homelessness, redundancy or being sacked from a job, extended work search without success, major financial crisis, something valued being lost or stolen, having trouble with the police involving court appearance, and serving time in prison.

##### Hazardous alcohol use

Hazardous alcohol use was operationalised as scoring 8 or above on the Alcohol Use Disorders Identification Test scale.^20^

##### Illicit drug use in the past year

Illicit drug use was based on use of cannabis, amphetamines, cocaine, crack, ecstasy, heroin, crystal methamphetamine, tranquilisers, amyl nitrate/poppers, anabolic steroids, glue/solvents/gas/aerosols, acid/LSD, and magic mushrooms.

#### Psychiatric morbidity

- The presence of common mental disorder (CMD) was derived from the Revised Clinical Interview Schedule, based on ICD-10 criteria for generalised anxiety disorder, depressive episode, phobia, obsessive compulsive disorder, panic disorder or CMD not otherwise specified, and binary coded into 0–11 and 12+.^21^
- Presence of post-traumatic stress disorder (PTSD) was ascertained using the PTSD Checklist, a self-report measure for probable PTSD.^22^ PTSD symptoms were defined based on endorsement of either DSM-IV criteria for PTSD in the previous month or a score of 50 or more on PTSD symptom domain scores for arousal, avoidance and re-experiencing phenomena.
- Psychosis was classified using the Psychosis Screening Questionnaire and defined as the endorsement of secondary questions on any of the domains.^23^

##### Non-partner violence and criminal justice involvement

Lifetime non-partner violence was defined as endorsement of items for having been in a fight which involved either children, non-partner family members, friends, some other person known to the respondent, a stranger, a police officer or some other person in the respondent's lifetime. Lifetime criminal justice involvement was defined as endorsement of being in trouble with the police which involved a court appearance or time in prison on remand or serving a sentence. These items were asked only of participants aged 16 to 64 years.

### Analysis

#### Objective 1: the association of lifetime IPVP with use of mental health services

Data were analysed in Stata 16. All regressions were based on data fully observed for the final modelled variables and incorporated survey weights. To test objective 1, we first specified a gender interaction term and included it in all models, applying linear combination to estimate odds ratios (ORs) with 95% confidence intervals among men and women. Separate models were used to estimate associations between lifetime IPVP and any mental health service use in the past year, counselling, GP consultation for a physical reason in the past year, GP consultation for a psychological or emotional problem in the past year, and lifetime psychiatric admission. In the first model (model I), we included age and sociodemographics (ethnic group, marital status, socioeconomic class, educational attainment and neighbourhood deprivation). In the second model (model II), we estimated models adjusting for IPV victimisation and other life adversities. Models were estimated based on the complete sample and then based on data from respondents without self-reported lifetime criminal justice involvement.

*O2. Assess whether associations with mental health service use were specific to IPVP or a reflection of general violent behaviour, by evaluating associations of a multiple category variable including non-partner violence only, IPVP only, and both non-partner violence and IPVP, with mental health service use.*

We estimated the association of the four-category violence variable (no violence, non-partner violence perpetration only, IPVP only, and both non-partner violence perpetration and IPVP) with mental health service use outcomes including covariate sets for models as described above. Models were estimated based on the overall data and then based on data from respondents without self-reported criminal justice involvement in their lifetime. As information on non-partner violence perpetration was only available in 16- to 64-year-olds, these models were based on smaller numbers of participants than those used for the other objectives.

#### Sensitivity analyses

We used multiple imputation to examine the possible impact of missing data on our results. Analyses of objectives 1 and 2 were re-estimated based on multiple imputation (of mental health service use, IPVP and explanatory variables) through chained equations, combining five imputed data-sets. The imputation model specified a logit link for all variables except for ethnicity (multinomial logit), educational attainment and social class (ordinal logit), and other life adversities (linear regression).

To assess the possible impact of misreporting of IPVP on our results, we did probabilistic sensitivity analysis using EPISENS in Stata 16.^24^ This procedure allows researchers to pre-specify plausible sampling values for sensitivity and specificity of the exposure or outcome and produces results combined from a series of replications. We specified the following scenarios with 2000 replications: low sensitivity for IPVP in both those with and those without mental health service use, maintaining high specificity; lower sensitivity in those with mental health service use than those without, with higher specificity; and a scenario involving reduced sensitivity and specificity for IPVP among those with and without mental health service use.

### Ethics approval

Ethical approval for APMS 2014 was obtained from the West London National Research Ethics Committee with reference number 14/LO/0411.

### Ethical standards

The authors assert that all procedures contributing to this work comply with the ethical standards of the relevant national and institutional committees on human experimentation and with the Helsinki Declaration of 1965, as revised in 2008. Verbal consent was obtained from all survey participants and witnessed and formally recorded; details are contained in the APMS documentation. The project was approved by the King's College London Psychiatry, Nursing and Midwifery Research Ethics Panel in 2019, under reference number LRS-18/19-10496.

### Consent to participate

Consistent with standard practice on official surveys of the general population, after provision of advance written information and face-to-face explanation of survey process and data uses, verbal consent for voluntary participation was obtained on the doorstep, witnessed and formally recorded.

### Consent for publication

NHS Digital provided permission for data use, which allows for publication of aggregated results. The views expressed in this publication are those of the authors and not necessarily those of the National Institute for Health Research (NIHR) or the Department for Health and Social Care (DHSC). The funder had no role in the study design, data collection, data analysis, data interpretation or writing of this paper.

## Results

### Description of study population

#### IPVP, sociodemographic factors and IPV victimisation

A total of 7546 household residents provided data, of which 3058 (a weighted percentage of 48.8%) were men and 4488 (51.2%) were women. The prevalence of reported lifetime IPVP was similar for men and women (8.0% and 8.6%, respectively; Table 1). Lifetime IPVP ranged from 2.1% among those aged 75 years and over to 11.3% among those aged 45–54 years. There was tenfold variation in the prevalence of IPVP by IPV victimisation status, with 2.8% of those without IPV victimisation reporting IPVP, compared with 28.1% of those reporting IPV victimisation. IPVP was higher among those with three or more other life adversities (11.1%) compared with those with fewer than three adversities (5.1%).

**Table 1:** Description of sample characteristics, including missing data. All proportions are weighted for the survey design

	Lifetime IPVP overall^a^		Non-partner violence perpetration only^b^		IPVP only		Both non-partner violence perpetration and IPVP		Row totals (overall *N* in category)
	Count	%	Count	%	Count	%	Count	%	
Age, years									
16–24	42	7.4	67	16.9	20	3.4	22	4.0	560
25–34	99	8.7	59	8.2	78	6.7	20	1.9	1035
35–44	105	9.1	34	3.2	86	7.4	19	1.7	1180
45–54	143	11.3	17	1.6	126	9.5	16	1.7	1294
55–64	121	9.9	10	0.7	113	9.2	8	0.8	1226
65–74	72	5.8	−	−	−	−	−	−	1189
75+	16	2.1	−	−	−	−	−	−	1062
Sex									
Male	226	8.0	143	9.5	134	6.1	48	2.7	3058
Female	372	8.6	44	2.0	289	8.7	37	1.3	4488
Education									
Degree	127	6.9	29	2.6	103	6.2	9	0.7	1800
Teaching, higher national diploma, nursing	52	10.0	14	5.9	34	8.3	10	3.0	610
A Level	108	9.2	57	8.8	82	7.2	18	2.5	1192
GCSE or equivalent	176	9.7	63	7.4	129	8.4	29	2.2	1747
Foreign/other	10	4.8	2	3.3	5	4.1	1	2.5	272
No qualifications	118	7.2	21	4.2	65	8.3	17	2.6	1843
Missing	8	11.2	1	1.5	5	10.7	1	3.4	82
Ethnicity									
White British	521	8.5	164	6.0	360	7.7	73	2.0	6387
White Other	28	8.4	10	4.6	23	7.1	4	1.9	426
Black African or Black Caribbean	21	12.0	5	5.2	17	10.0	3	2.8	197
Asian or Asian British	14	3.3	6	4.6	12	3.1	2	0.4	357
Mixed or multiple ethnicity	10	8.1	2	2.9	8	6.5	2	1.9	151
Missing	4	26.3	0	0.0	3	24.8	1	10.7	28
Neighbourhood deprivation^c^									
Least deprived (lowest score)	95	6.2	36	5.0	68	6.1	5	0.7	1554
2	89	5.8	38	5.8	62	5.2	15	1.5	1550
3	113	8.1	22	3.4	81	8.0	14	1.6	1563
4	131	10.2	45	6.6	99	8.6	20	3.0	1457
Most deprived (highest score)	170	11.4	46	7.2	113	8.9	31	2.8	1422
Socioeconomic class^d^									
Managerial and professional	150	8.2	47	3.7	131	7.4	9	0.6	1795
Intermediate occupation	63	9.3	32	5.5	50	6.8	12	2.7	678
Small employer	39	9.2	27	7.8	28	6.2	7	3.3	426
Lower supervisory	18	9.6	11	8.0	15	7.7	3	2.2	205
Semi-routine/routine	123	10.4	47	7.4	93	7.6	27	3.0	1137
Never worked/not working	186	7.0	22	2.7	92	9.2	21	2.1	2942
Not classified for other reasons	13	3.8	22	15.2	8	2.5	5	1.6	316
Missing	7	22.9	0	0.0	6	20.8	1	4.3	47
Marital status									
Married	280	7.5	78	3.5	206	7.0	34	1.5	4137
Single	152	9.6	97	12.4	109	6.4	40	3.3	1588
Widowed	25	3.0	2	1.5	7	4.3	1	0.6	853
Divorced	141	15.4	10	1.4	101	15.2	10	1.7	968
IPV victimisation^e^									
No	137	2.8	126	5.5	93	2.6	12	0.5	5088
Yes	455	28.1	61	6.2	327	22.3	73	6.5	1649
Missing	6	6.7	0	0.0	3	5.6	0	0.0	809
Other life adversities^f^									
Below three	163	5.1	83	5.5	128	4.7	15	0.2	3312
Three and above	435	11.1	104	5.8	295	10.1	70	0.4	4234
Common mental disorder^g^									
No	364	6.3	137	5.3	250	5.7	38	1.3	6209
Yes	234	18.1	50	7.2	173	14.8	47	4.9	1337
Psychosis^h^									
No	507	7.6	155	5.1	364	7	58	1.5	7087
Yes	91	20.0	32	13.5	59	12.6	27	8.6	459
PTSD^i^									
No	418	6.9	153	5.1	327	6.3	51	1.4	6521
Yes	180	25.1	33	11.2	96	18.9	34	7.4	540
Missing	0	0.0	1	18.3	0	0.0	0	0.0	485
Hazardous alcohol use^j^									
No	409	6.7	96	3.7	287	6.3	47	1.3	5965
Yes	188	14.7	91	12.3	135	11.0	38	4.2	1253
Missing	1	4.9	0	0.0	1	8.7	0	0.0	328
Drug use in the past year^k^									
No	462	7.1	117	3.9	336	6.7	44	1.3	6448
Yes	105	19.2	56	18.6	68	12.3	35	7.0	461
Missing	31	16.4	14	10.8	19	11.9	6	4.3	637
Lifetime involvement with criminal justice or the police^l^									
No	480	0.4	146	4.9	349	6.7	58	1.5	7017
Yes	114	2.1	41	14.7	71	15.5	26	7.2	503
Missing	4	28.5	0	0.0	3	21.1	1	10.6	26
**Health service use**									
Counselling									
No	558	8.0	175	5.5	400	7.3	72	1.8	7302
Yes	40	17.2	12	10.6	23	10.0	13	7.7	244
GP for a physical reason in past year									
No	196	7.4	93	7.1	144	6.0	33	2.1	2781
Yes	402	8.9	94	4.6	279	8.4	52	1.9	4763
GP for a mental health reason in past year									
No	408	6.8	155	5.7	285	6.1	45	1.4	6512
Yes	190	19.0	32	5.6	138	15.2	40	5.1	1031
Psychiatric admission									
No	558	8.0	183	5.7	401	7.2	75	1.8	7334
Yes	40	21.9	4	3.2	22	15.6	10	8.7	212
Any mental health service use in the past year									
No	63	6.7	155	5.7	285	6.1	44	1.4	6494
Yes	184	19.0	32	5.6	138	15.1	41	5.1	1044

a. Lifetime IPVP was defined as the endorsement of either threatening words/behaviour, physical violence or sexual violence towards a partner or ex-partner in the respondent's lifetime. Data for IPVP were missing for 774 respondents (10.3%).

b. Lifetime non-partner violence perpetration was defined as endorsement of items for having been in a fight which involved either children, non-partner family members, friends, some other person known to the respondent, a stranger, a police officer or some other person in the respondent's lifetime. This variable was only collected for those aged under 65 years, so analysis of the multiple category variable for IPVP and non-partner violence perpetration included only those under 65 (whereas the binary IPVP variable was collected for the entire sample). Data for the multiple category IPVP/non-partner violence perpetration variable were missing for 459 respondents (8.7%) under 65 (who were eligible for recording of this variable). There were 2251 respondents who were over 65 and were not eligible for measurement of this variable and were not included in models for this variable.

c. Neighbourhood deprivation was measured using the Index of Multiple Deprivations 2017.

d. Socioeconomic class was measured using the National Statistics Socio-Economic Classification.

e. IPV victimisation was defined as the experience of the following: experience of a partner preventing you from having a fair share of the household money; repeatedly belittling you to the extent that you felt worthless; pushing you, holding you, pinning you down or slapping; sending you more than one unwanted letter, email, text message or card that was either obscene or threatening and which caused you fear, alarm or distress; or kicking you, biting you, hitting you with a fist or something else, or throwing something at you that hurt you.

f. Number of other (non-IPV) lifetime adversities was derived based on binary items for ever having experienced: serious illness or injury, serious illness or injury to a close relative, serious assault of a close relative, death of an immediate family member, death of a close family friend or other relative, violence at work, homelessness, redundancy or being sacked from a job, extended work search without success, major financial crisis, something valued being lost or stolen, having trouble with the police involving court appearance or serving time in prison.

g. Common mental disorder was defined as a score of 12 or more on the Revised Comprehensive Interview Schedule.

h. Psychosis was measured using the Psychosis Screening Questionnaire and defined as the endorsement of secondary questions on any of the domains.

i. PTSD was defined based on endorsement of either DSM-IV criteria for PTSD in the previous month or exceeding a threshold of 50 on PTSD symptom domain scores for arousal, avoidance and re-experiencing phenomena.

j. Hazardous alcohol use was defined as scoring higher than 8 on the Alcohol Use Disorders Identification Test scale.

k. Due to a singleton stratum for past year drug use, these data were coded so that they did not contribute to standard errors.

l. Lifetime criminal justice involvement was defined as endorsement of being in trouble with the police which involved a court appearance, or time in time in prison on remand or serving a sentence.

#### IPVP, psychiatric morbidity and mental health service use

Higher proportions of IPVP were noted for those with all psychiatric morbidity and alcohol and/or drug use compared with those without, as evident for CMD (18.1% *v*. 6.3%), PTSD (25.1% *v*. 6.9%), psychosis (20% *v*. 7.6%), hazardous alcohol use (14.7% *v*. 6.7%) and past year drug use (19.2% *v*. 7.1%). The prevalence of IPVP was higher in those reporting counselling (17.2%), GP consultation for a physical reason in the past year (8.9%), GP consultation for a mental health reason in the previous year (19.0%), psychiatric admission (21.9%) or any mental health service use in the previous year (19.0%), compared with those not reporting these indicators.

#### Non-partner violence perpetration

Unlike IPVP, non-partner violence perpetration declined with age, ranging from 16.9% (in 16- to 24-year-olds), to 0.7% (in 55- to 64-year-olds). Non-partner violence perpetration was more common among men (9.5%) compared with women (2.0%). Similar patterns to those seen for IPVP were observed for the prevalence of non-partner violence with categories of neighbourhood deprivation, psychiatric morbidity and alcohol and drug use.

#### Association of IPVP with mental health service use

In models adjusting for age and sociodemographic variables, statistical evidence was found for associations between IPVP and any mental health service use in the past year (men: OR 2.8, 95% CI: 1.8–4.2; women: OR 2.8, 95% CI: 2.1–3.8), as well as GP consultation for a mental health reason in the previous year and psychiatric admission. These associations were also evident upon restricting to those not reporting criminal justice involvement (Table 2). Experience of IPV victimisation and other life adversities explained much of this association.

**Table 2:** Associations (odds ratios, ORs, with 95% confidence intervals in parentheses) of lifetime IPVP with health service use, including for mental health problems

	Data complete for analysed variables (*n* = 6448)		Among those with no criminal justice involvement (*n* = 6045)	
	IPVP (men)	IPVP (women)	IPVP (men)	IPVP (women)
Any mental health service use in the past year	I	2.8 (1.8–4.2)	2.8 (2.1–3.8)	2.9 (1.7–4.8)	2.3 (1.7–3.2)
	II	1.5 (1.0–2.4)	1.6 (1.1–2.2)	1.7 (1.0–3.0)	1.4 (1.0–2.0)
Counselling	I	1.8 (0.7–4.2)	2.3 (1.4–3.8)	1.7 (0.5–5.8)	1.4 (0.8–2.7)
	II	0.8 (0.3–2.3)	1.3 (0.8–2.2)	1.1 (0.3–4.0)	1.0 (0.5–1.9)
GP for a physical reason in the previous year	I	1.3 (0.9–1.8)	1.3 (1.0–1.7)	1.4 (0.9–2.2)	1.2 (0.9–21.7)
	II	1.0 (0.7–1.4)	1.0 (0.8–1.4)	1.2 (0.7–1.8)	1.0 (0.7–1.4)
GP for a mental health reason in the past year	I	2.8 (1.8–4.3)	2.8 (2.1–3.7)	2.9 (1.7–4.8)	2.3 (1.7–3.2)
	II	1.5 (1.0–2.5)	1.6 (1.1–2.2)	1.7 (1.0–3.0)	1.4 (1.0–2.0)
Psychiatric admission	I	4.0 (2.2–7.3)	2.4 (1.3–4.4)	3.9 (1.4–10.9)	1.8 (0.8–3.8)
	II	2.2 (1.1–4.4)	1.4 (0.8–2.6)	2.4 (0.8–7.5)	1.1 (0.5–2.4)

Model I: adjusted for age and sociodemographic variables (which were educational attainment, ethnic group, neighbourhood deprivation, socioeconomic class and marital status).

Model II: adjusted for age, sociodemographic variables, IPV victimisation and other life adversities.

Fully adjusted estimates for association of IPVP for all mental health service use indicators had confidence intervals crossing null, suggesting no significant associations after accounting for the explanatory variables.

#### IPVP and general violence

Analysing the association of a four-level categorical variable (no violence, non-partner violence perpetration only, IPVP only, and both non-partner violence perpetration and IPVP) allowed consideration of whether association of IPVP with mental health service use indicators was a reflection of generally violent behaviour (Table 3). Statistically significant associations were found between IPVP only and any mental health service use in the past year and GP consultation for a mental health reason in the past year. Similar to our findings for the binary IPVP variable, adjustment for sociodemographic variables had a limited impact on estimates; however, adjustment for IPV victimisation and other life adversities substantially attenuated estimates towards null, and final estimates indicated limited statistical evidence for association of IPVP with any indicator of mental health service use in either men or women. Restricting analyses to those without criminal justice involvement strengthened associations among men and weakened associations among women, although confidence intervals overlapped. We found no evidence for association of IPVP with indicators of mental health service use among those without criminal justice involvement after adjustment.

**Table 3:** Associations of IPVP and non-partner violence perpetration with health service use, including for mental health problems

	All data (*n* = 4681)						Only those without criminal justice involvement (*n* = 4275)					
	Men			Women			Men			Women		
	Non-partner violence perpetration only	IPVP only	Both non-partner violence perpetration and IPVP	Non-partner violence perpetration only	IPVP only	Both non-partner violence perpetration and IPVP	Non-partner violence perpetration only	IPVP only	Both non-partner violence perpetration and IPVP	Non-partner violence perpetration only	IPVP only	Both non-partner violence perpetration and IPVP
Any mental health service use in the past year	I	1.2 (0.6–2.4)	2.8 (1.6–4.8)	3.4 (1.6–7.1)	2.1 (0.9–4.7)	2.5 (1.8–3.5)	5.6 (2.6–12.3)	1.0 (0.4–2.4)	2.9 (1.5–5.6)	3.6 (1.4–9.2)	2.6 (1.1–5.9)	2.1(1.4–3.0)	4.4 (1.8–10.8)
	II	0.9 (0.5–1.8)	1.7 (1.0–2.8)	2.0 (1.0–4.3)	1.6 (0.7–3.9)	1.5 (1.1–2.2)	2.6 (1.2–5.9)	0.9 (0.4–2.0)	1.8 (1.0–3.5)	2.1 (0.7–6.0)	2.1 (0.8–5.2)	1.3 (0.9–1.9)	2.1 (0.8–5.7)
Counselling	I	3.5 (1.3–9.4)	0.8 (0.3–2.5)	5.8 (1.6–21.9)	1.1 (0.3–4.8)	1.6 (0.8–2.9)	7.3 (2.6–20.6)	3.9 (1.3–12.1)	0.7 (0.2–3.3)	6.5 (1.2–35.6)	1.4 (0.3–6.1)	1.2 (0.5–2.6)	2.4 (0.6–10.0)
	II	2.4 (1.0–6.2)	0.5 (0.2–1.5)	2.1 (0.4–11.6)	0.7 (0.1–3.6)	1.0 (0.5–2.0)	3.0 (1.1–8.3)	3.5 (1.2–10.5)	0.5 (0.1–2.5)	4.7 (0.7–30.9)	1.0 (0.2–5.0)	0.9 (0.4–2.1)	1.4 (0.3–6.4)
GP for a physical reason in the past year	I	1.1 (0.7–1.7)	1.4 (0.9–2.3)	1.1 (0.6–2.3)	1.1 (0.5–2.3)	1.2(0.8–1.6)	1.4 (0.7–3.2)	1.0 (0.6–1.7)	1.6 (0.9–2.9)	1.4 (0.6–3.1)	1.3 (0.6–2.8)	1.1 (0.8–1.6)	1.3 (0.5–3.3)
	II	0.9 (0.6–1.4)	1.1 (0.7–1.7)	0.9 (0.4–1.7)	1.0 (0.5–2.1)	1.0(0.7–1.4)	1.0 (0.5–2.3)	1.0 (0.6–1.6)	1.2 (0.7–2.4)	1.2 (0.5–2.7)	1.1 (0.5–2.5)	1.0 (0.7–1.4)	1.0 (0.4–2.4)
GP for a mental health reason in the past year	I	1.2 (0.6,2.4)	2.9(1.7–4.9)	3.5 (1.7–7.2)	2.1 (1.0–4.7)	2.5(1.8–3.5)	5.3 (2.4–11.6)	1.0 (0.4–2.4)	2.9 (1.5–5.6)	3.6 (1.4–9.2)	2.6 (1.1–5.9)	2.1 (1.5–3.0)	4.0 (1.6–10.0)
	II	0.9 (0.5–1.8)	1.7 (1.0–2.8)	2.0 (1.0,4. 3)	1.7 (0.7–4.0)	1.5 (1.1–2.2)	2.5 (1.1–5.6)	0.9 (0.4–2.0)	1.8 (1.0–3.5)	2.1 (0.7–6.0)	2.1 (0.8–5.2)	1.3 (0.9–1.9)	1.9 (0.7–5.3)
Psychiatric admission	I	1.3 (0.3–5.1)	2.4 (0.9–6.1)	9.0 (3.8–21.6)	1.3 (0.3–5.1)	2.0 (0.9–4.2)	5.6 (1.6–20.2)	1.1 (0.2–5.2)	3.7 (1.3–10.5)	5.6 (0.5–57.8)	1.1 (0.2–5.2)	1.6(0.6–4.2)	2.5 (0.5–12.1)
	II	1.0 (0.3–2.9)	1.3 (0.5–3.4)	3.3 (1.1–9.9)	1.0 (0.3–2.9)	1.4 (0.7–3.0)	2.3 (0.6–8.1)	0.9 (0.2–4.6)	2.4 (0.8–7.1)	3.6 (0.2–54.0)	0.9 (0.2–4.6)	1.1 (0.4–2.8)	1.2 (0.2–7.0)

Model I: adjusted for age and sociodemographic variables (educational attainment, ethnic group, neighbourhood deprivation, socioeconomic class and marital status).

Model II: adjusted for age, sociodemographic variables, IPV victimisation, and other life adversities.

Reference group for all estimates is those with no reported violence (neither non-partner violence perpetration nor IPVP)

#### Sensitivity analyses

Multiple imputation analyses did not indicate differences in our inference for either the binary IPVP variable (Supplementary Table 1 available at https://doi.org/10.1192/bjo.2023.51) or the multiple category variable for non-partner violence perpetration, IPVP only, and both non-partner violence perpetration and IPVP (Supplementary Table 2). Probabilistic sensitivity analyses for IPVP misclassification suggested that all misclassification scenarios we specified generated median estimates which were more extreme (further from null) than our main results (Supplementary Table 3).

## Discussion

### Summary of findings

Self-reported IPVP is common in mental health service users, with a prevalence of around 8% in men and women. We found strong associations between self-reported IPVP and mental health service use, including current use of counselling, seeing a GP for a mental health reason in the previous year, lifetime psychiatric admission and any mental health service use in the previous year (objective 1). This was evident in people only reporting IPVP without non-partner violence perpetration and among those not reporting criminal justice involvement (objective 2). These associations were not fully explained by socioeconomic or demographic variables but were considerably attenuated when account was taken of IPV victimisation and other life adversities. We did not find evidence for differences between men and women in the association of IPVP with mental health service use, including when stratifying into IPVP only and IPVP with non-partner violence perpetration.

### Interpretation

Similar proportions of men and women reported IPVP; however, study items did not capture information on severity and frequency of violence, which may vary significantly between men and women according to previous evidence.^5^ Although IPVP is relatively common among people using mental health services, a substantial proportion of people reporting IPVP who also report mental health service use are not identified or assessed in criminal justice settings; health professionals could therefore be important for contact with this group, which may be otherwise hard to reach. The association of IPVP with mental health service use may be strongly explained by life adversities, underlining the need to identify and address adversities including IPV victimisation, as well as addressing the safety of the patient, their partners (and ex-partners) and children.

Our findings have implications for risk assessment in mental health services, which is an ongoing focus for policy improvement.^25^ The literature suggests that IPVP is associated with a risk of non-intimate partner violence. For example, in a US study, 36% of IPV victims described their partner as also violent towards non-family members.^26^ A Dunedin birth cohort analysis found a strong association between IPVP and violence towards strangers.^27^ Delsol found that perpetrators of IPV who also reported non-partner violence perpetrated most violence *outside* the intimate relationship.^28^ The possible risk of further violence towards others posed by perpetrators of IPV is an important concern which has driven research into risk assessment in the context of IPVP.^29^ Our finding of a significant proportion of self-reported IPVP among people recently using mental health services underscores the need to understand the effectiveness and impact of risk assessment procedures when IPVP is identified in mental health clinical practice. We did not find that the association of IPVP with mental health service use was accounted for by non-partner violence perpetration, in either men or women.

There is evidence that specialist behavioural management programmes may be effective for IPVP in the general population;^30^ however, their effectiveness among people with mental health conditions is not yet known. In some people with mental health conditions, risk of violence towards others may be reduced by consistent treatment.^31^ This implies that it might be possible to develop refined interventions to reduce risk of IPVP in people with mental health conditions.

### Previous findings on this topic

A relationship between IPVP and a range of mental health conditions has been established, but few studies have examined mental health service use. In a systematic review of the association between mental health conditions and IPVP by Oram et al, no included studies examined mental health service use in a representative national general population sample.^32^ Being arrested for IPVP was associated with secondary mental health service use in Swedish men.^13^ This analysis offers an overlapping perspective, as we used a self-reported measurement of IPVP rather than a criminal justice measure. Hester et al^33^ found that CMD was more common among men reporting negative relationship behaviours, but this was among a study population of GP attenders.

### Strengths and limitations

We used nationally representative data, with results which are generalisable to the National Health Service (NHS) in England. There have been very few epidemiological examinations of the relationship between IPVP and mental health service use using nationally representative data that allow the assessment of alternative explanations (including other predictors of need, such as IPV and other life adversities). In comparison with the study of Yu et al on IPV arrest,^13^ our investigation of national survey data probably captured some IPVP which would not have been ascertained through criminal justice routes. Although Yu et al were able to take account of genetic relatedness through a sibling comparison design, that analysis did not evaluate the impact of IPV victimisation or other life adversities. Our study also allowed assessment of associations separately for men and women. Therefore, our study presents an important contribution to our understanding of the mental health service response towards IPVP.

Key limitations are that self-report IPVP probably did not capture all IPVP, potentially owing to social undesirability affecting reporting,^34^ or the gendered differences in severity and impact of IPVP. However, our results were not affected by missing data (assuming data were missing at random), and quantitative bias analysis suggested that most reasonable patterns of misclassification of IPVP were unlikely to change our results. Our results cannot inform the question of a causal relationship between mental disorders and IPVP. The impact of adjusting for IPV victimisation on our results could be because of the tendency of IPV perpetrators to inaccurately report that they have been exposed to IPV victimisation. Although our study was conducted using data collected before the pandemic, we expect that the direction of the associations are generalisable to the current situation in England. This needs further study, including in the context of future national surveys of psychiatric morbidity in England and elsewhere.

### Implications

There is a large excess prevalence of IPVP among both men and women using mental health services. This is evident even among people who do not report contact with the criminal justice system, indicating that non-criminal justice settings could play an important part in responding to IPVP. The association of IPVP with mental health service use is partly explained by self-reported IPV victimisation and other life adversities, indicating that these characteristics may be relevant for identifying IPVP in mental health services. Our findings underline the need for mental health services to develop evidence-based interventions to improve identification and response to IPVP and reduce risks to patients and family members (including family carers). Based on the relatively high prevalence of IPVP in mental health services, these services may also provide an important site for onward referral of perpetrators of IPV to specialised perpetrator programmes.

## Data Availability

Requests for access to the 2014 data-set should be made to the Data Access Request Service at NHS Digital. Survey data collection was programmed in Blaise, a computer-assisted interviewing system and survey processing tool for the Windows operating system. The system was developed by Statistics Netherlands and was designed for use in official statistics. It is available to National Statistical Institutes and related research institutes. Data management and analysis were conducted in Stata 16, and the syntax is available from the authors on request.

## Supplementary material

For supplementary material accompanying this paper visit http://doi.org/10.1192/bjo.2023.51. click here to view supplementary material
