# Supplementary material for: Intimate partner violence perpetration and mental health service use in England: analysis of nationally representative survey data
Source: BJPsych Open. 2023 Apr 12;9(3):e64. doi: 10.1192/bjo.2023.51 (PMC10134318; doi:10.1192/bjo.2023.51)
Supplement: Supplementary file 1 [file S2056472423000510sup001.docx]

| ­­Supplementary Table 1. Imputed estimates for association (odds ratios(OR), with lower and upper 95% confidence intervals, LCI and UCI respectively) of IPVP with mental health service use. Estimates were combined from 5 imputed datasets from 7546 respondents. | | | | | | | | | | | | | |
| --- | --- | --- | --- | --- | --- | --- | --- | --- | --- | --- | --- | --- | --- |
|  | | All data - men | | | All data - women | | | No criminal justice involvement - men | | | No criminal justice involvement- women | | |
|  |  | OR | LCI | UCI | OR | LCI | UCI | OR | LCI | UCI | OR | LCI | UCI |
| Counselling | I | 1.9 | 0.7 | 4.7 | 2.2 | 1.4 | 3.5 | 1.8 | 0.6 | 5.5 | 1.5 | 0.8 | 2.7 |
|  | II | 0.9 | 0.3 | 2.5 | 1.2 | 0.7 | 1.9 | 1.0 | 0.3 | 3.5 | 0.9 | 0.5 | 1.7 |
|  |  |  |  |  |  |  |  |  |  |  |  |  |  |
| Seen GP for a physical reason in past year | I | 1.3 | 0.9 | 1.9 | 1.4 | 1.0 | 1.8 | 1.4 | 0.9 | 2.2 | 1.3 | 0.9 | 1.8 |
|  | II | 1.0 | 0.7 | 1.4 | 1.1 | 0.8 | 1.5 | 1.2 | 0.8 | 1.8 | 1.0 | 0.8 | 1.4 |
|  |  |  |  |  |  |  |  |  |  |  |  |  |  |
| Seen GP for a mental health reason in the past year | I | 3.2 | 2.1 | 4.7 | 2.8 | 2.1 | 3.8 | 3.3 | 2.0 | 5.6 | 2.4 | 1.7 | 3.3 |
|  | II | 1.7 | 1.1 | 2.5 | 1.5 | 1.1 | 2.1 | 1.0 | 1.2 | 3.3 | 1.4 | 1.0 | 2.0 |
|  |  |  |  |  |  |  |  |  |  |  |  |  |  |
| Psychiatric admission | I | 3.5 | 1.9 | 6.3 | 2.4 | 1.4 | 4.1 | 3.7 | 1.4 | 9.9 | 1.9 | 1.0 | 3.7 |
|  | II | 1.8 | 1.0 | 3.5 | 1.3 | 0.7 | 2.3 | 2.3 | 0.9 | 6.4 | 1.2 | 0.6 | 2.3 |
|  |  |  |  |  |  |  |  |  |  |  |  |  |  |
| Any mental health service use in the past year | I | 3.2 | 2.2 | 4.6 | 2.8 | 2.1 | 3.8 | 3.4 | 2.1 | 5.4 | 2.4 | 1.8 | 3.4 |
|  | II | 1.7 | 1.1 | 2.5 | 1.6 | 1.1 | 2.2 | 2.0 | 1.3 | 3.3 | 1.4 | 1.0 | 2.0 |
|  |  |  |  |  |  |  |  |  |  |  |  |  |  |
| Model I: adjusted for age and sociodemographic variables (which were educational attainment, ethnic group, neighbourhood deprivation, socioeconomic class, and marital status).  Model II: adjusted for age, sociodemographic variables, IPV victimisation and other life adversities. | | | | | | | | | | | | | |

| Supplementary Table 2: Imputed estimates for multiple category violence exposure. | | | | | | | | | | | | | |
| --- | --- | --- | --- | --- | --- | --- | --- | --- | --- | --- | --- | --- | --- |
|  |  | All data | | | | | | No criminal justice involvement | | | | | |
|  |  | Men |  |  | Women |  |  | Men |  |  | Women |  |  |
|  |  | Non-partner violence perpetration only | IPVP only | Both non-partner violence perpetration and IPVP | Non-partner violence perpetration only | IPVP only | Both non-partner violence perpetration and IPVP | Non-partner violence perpetration only | IPVP only | Both non-partner violence perpetration and IPVP | Non-partner violence perpetration only | IPVP only | Both non-partner violence perpetration and IPVP |
| Counselling | I | 3.0(1.2,7.5) | 0.9(0.3,2.3) | 4.6(1.3,16.7) | 1.0(0.2,4.3) | 1.6(0.9,2.9) | 7.1(2.8,17.9) | 3.7(1.3, 11.0) | 0.7(0.1,2.9) | 6.0(1.1,32.2) | 1.2(0.3,5.3) | 1.2(0.5,2.5) | 2.5(0.6,10.5) |
|  | II | 2.5(1.0,6.2) | 0.6(0.2,1.6) | 2.0(0.4,11.4) | 0.7(0.1,3.4) | 1.1(0.6,2.1) | 2.9(1.0,8.1) | 3.2(1.1,9.5) | 0.5(0.1,2.3) | 3.2(0.4,24.7) | 0.9(0.2,4.5) | 0.9(0.4,1.9) | 1.3(0.3,5.7) |
|  |  |  |  |  |  |  |  |  |  |  |  |  |  |
| Seen GP for a physical reason in the past year | I | 1.0(0.7,1.5) | 1.4(0.9,2.1) | 1.1(0.6,2.1) | 1.2(0.5,2.4) | 1.2(0.9,1.7) | 1.5(0.7,3.3) | 0.9(0.6,1.5) | 1.6(1.0,2.8) | 1.4(0.6,3.0) | 1.3(0.6,2.9) | 1.2(0.8,1.6) | 1.4(0.6,3.3) |
|  | II | 0.9(0.6,1.4) | 1.2(0.8,1.8) | 0.9(0.5,1.7) | 1.0(0.5,2.1) | 1.0(0.7,1.4) | 1,.0(0.5,2.3) | 0.9(0.5,1.4) | 1.4(0.8,2.3) | 1.1(0.5,2.4) | 1.1(0.5,2.6) | 1.0(0.7,1.4) | 1.0(0.4,2.5) |
|  |  |  |  |  |  |  |  |  |  |  |  |  |  |
| Seen GP for a mental health reason in the past year | I | 1.2(0.6,1.2) | 2.9(1.8,4.7) | 3.9(2.0,7.5) | 2.2(1.0,4.9) | 2.5(1.8,3.5) | 6.1(2.8,13.2) | 1.1(0.5,2.4) | 3.3(1.8,6.1) | 4.2(1.8,10.1) | 2.7(1.2,6.1) | 2.1(1.5,3.0) | 5.0(2.0,12.2) |
|  | II | 1.0(0.5,1.9) | 2.2(1.3,3.7) | 3.0(1.5,6.1) | 1.6(0.7,3.9) | 1.5(1.1,2.2) | 2.4(1.1,5.5) | 0.9(0.4,2.0) | 2.2(1.2,4.1) | 2.4(0.9,6.2) | 2.2(0.9,5.2) | 1.3(0.9,2.0) | 2.2(0.8,5.9) |
|  |  |  |  |  |  |  |  |  |  |  |  |  |  |
| Psychiatric admission | I | 1.2(0.4,4.0) | 1.9(0.8,4.5) | 6.0(2.5,14.2) | 1.2(0.4,4.0) | 1.9(0.9,3.9) | 5.1(1.8,14.5) | 0.9(0.2,4.0) | 2.8(1.0,7.8) | 4.5(0.5,44.4) | 0.9(0.2,4.0) | 1.7(0.7,4.0) | 2.6(0.6,12.2) |
|  | II | 0.9(0.3,2.8) | 1.3(0.5,3.3) | 3.6(1.3,9.9) | 0.9(0.3,2.8) | 1.4(0.7,2.9) | 2.3(0.6,8.3) | 0.8(0.2,3.6) | 2.3(0.8,6.5) | 2.8(0.2,37.1) | 0.8(0.2,3.6) | 1.4(0.6,3.3) | 1.4(0.3,7.5) |
|  |  |  |  |  |  |  |  |  |  |  |  |  |  |
| Any mental health service use in the past year | I | 1.2(0.6,2.2) | 2.9(1.8,4.7) | 3.8(2.0,7.5) | 2.2(1.0,4.8) | 2.5(1.8,3.4) | 6.5(3.0,13.9) | 1.1(0.5,2.4) | 3.3(1.8,6.1) | 4.3(1.8,10.1) | 2.6(1.2,6.0) | 2.1(1.5,3.0) | 5.4(2.2,13.2) |
|  | II | 0.9(0.5,1.8) | 1.7(1.0,2.8) | 2.0(0.9,4.2) | 1.6(0.7,3.9) | 1.5(1.1,2.2) | 2.6(1.1,5.8) | 0.9(0.4,2.0) | 2.2(1.2,4.1) | 2.4(0.9,6.2) | 2.1(0.9,5.1) | 1.3(0.9,1.9) | 2.4(0.9,6.4) |
|  |  |  |  |  |  |  |  |  |  |  |  |  |  |

| Supplementary Table 3. Probabilistic sensitivity analyses for the influence of misclassification of IPVP (exposure) on the measured association between IPVP and any mental health service use in the past year. Each set of estimates is based on 2000 replications. | | | |
| --- | --- | --- | --- |
|  | Percentiles | | |
|  | 2.5 | 50 | 97.5 |
| *Modelling measurement bias sampling from trapezoidal distributions for sensitivity and specificity in cases/non-cases, with low sensitivity (minimum 0.25, mode 0.35 - 0.55, and maximum 0.65) non-differential between cases and non-cases, with maintained non-differential specificity (min 0.75, mode 0.85 - 0.95, max 1).* | | | |
| Conventional | 2.82 | 3.41 | 4.11 |
| Systematic error | *4.84* | *11.42* | *192.52* |
| Systematic and random error | *4.71* | *11.52* | *199.14* |
| *Modelling measurement bias sampling from trapezoidal distributions for sensitivity and specificity in cases/non-cases, with lower sensitivity in cases (min 0.2, mode 0.3-0.5, max 0.6) compared to non-cases (min 0.3, mode 0.4-0.6, max 0.7), with non-differential specificity between cases and non-cases (min 0.75, mode 0.85 - 0.95, max 1).* | | | |
| Conventional | 2.82 | 3.41 | 4.11 |
| Systematic error | *6.33* | *17.58* | *330.92* |
| Systematic and random error | *6.17* | *17.75* | *334.66* |
| *Modelling measurement bias sampling from trapezoidal distributions for equally reduced sensitivity and specificity for both cases and non-cases (all min 0.65, mode 0.75 - 0.85, max 0.95).* | | | |
| Conventional | 2.82 | 3.41 | 4.11 |
| Systematic error | *10.95* | *24.38* | *430.29* |
| Systematic and random error | *10.58* | *24.53* | *494.79* |
